# Prognostic Significance of Bcl-2 and p53 Protein Expressions and Ki67 Proliferative Index in Diffuse Large B-cell Lymphoma

**DOI:** 10.4274/Tjh.2011.0034

**Published:** 2013-09-05

**Authors:** Betül Bolat Küçükzeybek, Sadi Bener, Aylin Orgen Çallı, Tuğba Doğruluk Paksoy, Bahriye Payzin

**Affiliations:** 1 Department of Pathology, İzmir Atatürk Training and Research Hospital, İzmir, Turkey; 2 Department of Pathology, Kahramanmaraş Yenişehir Government Hospital, Kahramanmaraş, Turkey; 3 3Department of Hematology, İzmir Atatürk Training and Research Hospital, İzmir, Turkey

**Keywords:** Diffuse large B-cell lymphoma, p53, Bcl-2, Ki67, prognosis

## Abstract

**Objective:** Diffuse large B-cell lymphoma (DLBCL) is a high-grade neoplasm that has heterogeneous properties in clinical, morphological, and immunophenotypic aspects. In the present study the effects of p53, Bcl-2, and Ki67 on prognosis and their relationships with clinical parameters were examined.

**Materials and Methods:** Thirty-five patients who had been diagnosed with nodally located DLBCL at İzmir Atatürk Training and Research Hospital between January 1999 and June 2006 were included in the study. The Ann Arbor classification system was used to determine the stage of the patients. The patients were evaluated according to age, sex, stage, B symptoms, extranodal involvement, and lactate dehydrogenase (LDH) level as well as immunohistochemically. P53 protein and Bcl-2 oncoprotein expressions and Ki67 proliferation index were assessed immunohistochemically.

**Results:** High Bcl-2 expression was found in 9 patients (25.7%), high p53 expression was found in 10 patients (28.6%), and high Ki67 was observed in 23 patients (65.7%). There was no significant correlation between p53 expression, Bcl-2 expression, or Ki67 proliferation index and age, sex, stage, B symptoms, extranodal involvement, LDH level, and overall survival (p>0.05). We did not find a relationship among p53 expression, Bcl-2 expression, Ki67 proliferation index, and prognosis (p>0.05). There was no significant relationship between overall survival and age, sex, stage, B symptoms, extranodal involvement, or LDH level (p>0.05). Our results revealed that Bcl-2 and p53 protein expressions and Ki67 proliferation index have no effect on overall survival of patients with DLBCL.

**Conclusion:** The prognostic importance of p53 and Bcl-2 protein expressions and Ki67 proliferation index in DLBCL, which has biological and clinical heterogeneity, can be understood in a large series of studies that have subclasses and immunohistochemical markers with optimal cut-off values.

## Abstract (tr)

**Amaç:** Diffuz büyük hücreli B lenfoma (DBBHL) klinik, morfolojik, immunofenotipik ve genetik özellikleri ile heterojenite gösteren yüksek dereceli bir neoplazmdır. Çalışmamızda DBBHL’da hücre siklusu düzenleyicisi olan p53 (tümör supresör gen), apoptozisi inhibe edici onkoprotein olan Bcl-2 ve hücre proliferasyon belirleyicisi olan Ki67 ekspresyonlarının klinik parametrelerle ilişkisini ve prognoz üzerindeki etkilerini araştırdık.

**Gereç ve Yöntemler:** Çalışmaya Ocak 1999 - Haziran 2006 tarihleri arasında, İzmir Atatürk Eğitim ve Araştırma Hastanesi Patoloji Bölümü’nde, nodal yerleşimli DBBHL tanısı alan 35 olgu alındı. Hastaların evrelemesinde Ann Arbor sınıflaması kullanıldı. Olgular yaş, cinsiyet, evre, B semptomları, ekstranodal tutulum, LDH düzeyi ve sağ kalımları yanı sıra immünohistokimyasal olarak; p53 protein ekspresyonu, Bcl-2 onkoprotein ekspresyonu ve Ki67 proliferasyon indeksi açısından değerlendirildi.

**Bulgular:** Yüksek Bcl-2 ekspresyonu 9 hastada (%25.7), yüksek p53 ekspresyonu 10 hastada (%28.6), yüksek Ki67 ekspresyonu 23 hastada (%65.7) saptandı. p53 ekspresyonu, Bcl-2 protein ekspresyonu ve Ki67 proliferasyon indeksi ile yaş, cinsiyet, evre, B semptomları, ekstranodal tutulum, LDH düzeyi ve sağ kalım arasında istatistiksel olarak anlamlı ilişki saptanmadı (p>0.05). Tüm olgularda p53, Bcl-2 ve Ki67 ekspresyonları ile prognoz arasındaki ilişki istatistiksel olarak anlamlı değildi (p>0.05). Sağ kalım süresi ile yaş, cinsiyet, evre, B semptomları, ekstranodal tutulum ve LDH düzeyi arasında istatistiksel olarak anlamlı ilişki bulunmadı (p>0.05). Bu çalışmada Bcl-2 ve p53 protein ekspresyonları ile Ki67 proliferasyon indeksinin, DBBHL’lı hastaların yaşam süresi üzerinde etkili olmadığı bulunmuştur.

**Sonuç:** p53 ve Bcl-2 protein ekspresyonları ile Ki67 proliferasyon indeksinin, DBBHL’daki prognostik önemi, immunohistokimyasal markerların optimal cut-off değerlerinin belirlendiği, subgrup ayrımının yapıldığı daha geniş serili çalışmalarda, biyolojik ve klinik heterojenitesi olan bu hastalıkta, daha net anlaşılacaktır. Anahtar Sözcükler: Diffüz büyük B hücreli lenfoma, p53, Bcl-2, Ki67, Prognoz

## INTRODUCTION

Diffuse large B-cell lymphoma (DLBCL) is the most common type of lymphoma, comprising 30%-40% of all non-Hodgkin’s lymphoma (NHL). It has heterogeneous clinical features and varies markedly in response to treatment and in prognosis [1,2]. The response rate is 60%-80% in NHL after acceptable therapy. The 5-year overall survival rate is higher than 55%. Patients who responded to chemotherapy and were cured after therapy could be estimated using clinic and laboratory results. Therapy plans can be determined using these prognostic factors [3,4]. Although survival can be estimated on the basis of clinical parameters, molecular abnormalities in a panel of suppressor proteins and oncogenic proteins have also been reported related with prognosis [5,6,7]. In the present study we investigated the effects of p53 (tumor suppressor protein), which is a cell cycle regulator; Bcl-2 oncoprotein, which is an inhibitor of apoptosis; and Ki67, which is a cell proliferation marker on prognosis, and their relationships with clinical parameters. 

## MATERIALS AND METHODS

**Figure 1:**
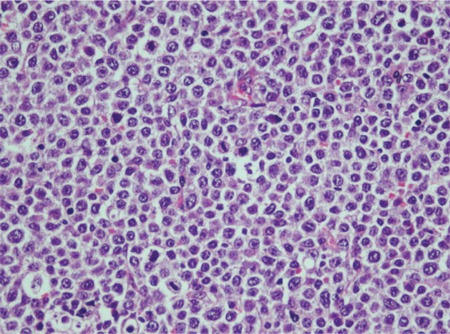
Morphologic features of DLBCL (hematoxylin and eosin, 440x).

Patients: Thirty-five patients who had been diagnosed with nodally located DLBCL at İzmir Atatürk Training and Research Hospital between January 1999 and June 2006 were included in the study. The patients were evaluated according to age, sex, stage, B symptoms, extranodal involvement, and lactate dehydrogenase (LDH) level as well as immunohistochemically. p53 and Bcl-2 oncoprotein expressions and Ki67 proliferation index were assessed immunohistochemically. The clinical parameters and the outcome were reviewed retrospectively. All clinical and laboratory data, along with the follow-up data, were obtained from the hospital records or patients’ charts. Overall survival (OS) was calculated from the date of diagnosis until death or last follow-up. Affected lymph nodes from all of the patients were examined by biopsy (Figure 1) and lymphoma was diagnosed according to WHO lymphoma classification. 

Immunohistochemistry: Paraffin sections were immunostained by the labeled streptavidin-avidin-biotin method with the antibodies for Ki67 (clone SP6, Neomarkers), Bcl-2 (clone 124, Dako), and p53 (clone DO7, Dako). Positive staining of small reactive lymphocytes for Bcl-2 was provided as an internal control. A previously known positive case was used as an external control in order to evaluate p53 reactivity. A known positive control (normal tonsil) was used to evaluate Ki67 reactivity. 

Three categories were defined for Bcl-2 [5,6,8,9] and p53 [6,7,8,10] expressions: negative when none or less than 10% of tumor cells showed staining, low expression when 10%-50% of tumor cells showed staining, and high expression when >50% of tumor cells showed staining. The cases were divided into 2 groups: a high Ki67 expression (>40%) group and a low Ki67 expression (<40%) group [1,11]. 

Statistical Analysis: Survival curves were drawn according to the Kaplan-Meier method and compared by log-rank test. The relationship between p53, Bcl-2, and Ki67 expressions and clinicopathological parameters was evaluated by chi-square test for data qualification and Fisher’s exact test for data categorization. Differences were considered as significant if the p value was less than 0.05. The study was approved by the ethics committee. 

## RESULTS

Patient characteristics are summarized in Table 1. Follow-up duration ranged from 0.5 to 68 months, with an average of 17.6 months. Fifteen (42.9%) patients were followed until death, whereas 20 (57.1%) patients were still alive at the end of the study. There were 21 (60%) men and 14 (40%) women in the study. The average age of the patients was 53.6 years. All patients were classified according to the Ann Arbor classification. As such, 12 patients (34.3%) had stage 1-2 and 23 patients (65.7%) had stage 3-4 disease. Elevated serum LDH levels were observed in 26 patients (74.3%). Eighteen (51.4%) patients had B symptoms at the time of diagnosis. Thirteen (37.1%) patients presented with involvement of extranodal sites. 

**Figure 2:**
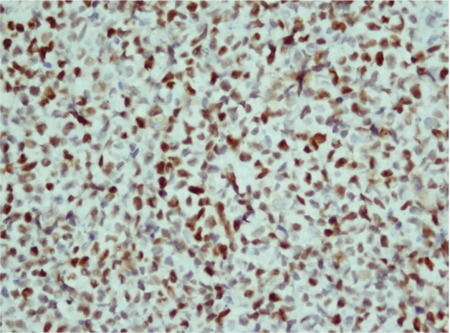
p53 nuclear positivity of >50% (440x).

**Figure 3:**
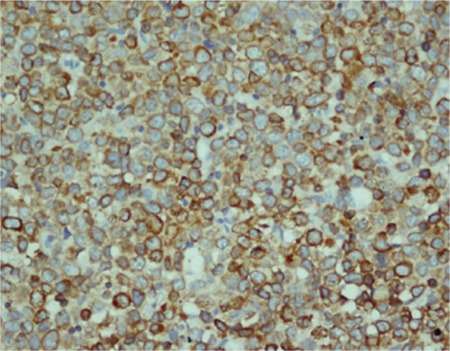
Bcl-2 cytoplasmic positivity of >50% (440x)

**Figure 4:**
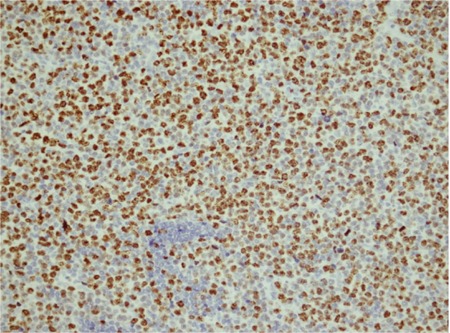
Ki67 nuclear positivity of >40% (220x)

p53 protein expression was high in 10 (28.6%) patients (Figure 2), low in 12 (34.3%) patients, and negative in 13 (37.1%) patients. Bcl-2 protein expression was high in 9 (25.7%), low in 4 (11.4%), and negative in 22 (62.9%) of the 35 cases (Figure 3). Ki67 expression was high in 23 (65.7%) patients and low in 12 (34.3%) patients (Figure 4). 

**Figure 5:**
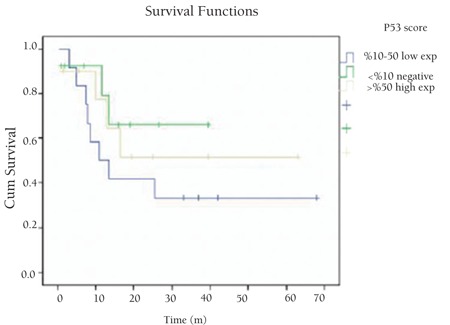
Overall survival related to p53 expression

**Figure 6:**
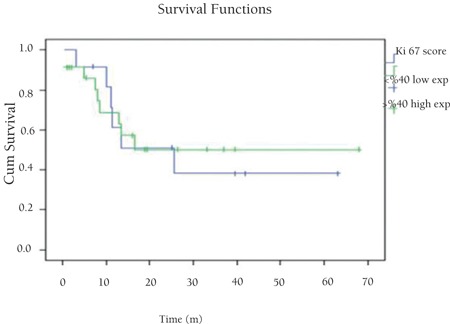
Overall survival related to Ki67 expression

**Figure 7:**
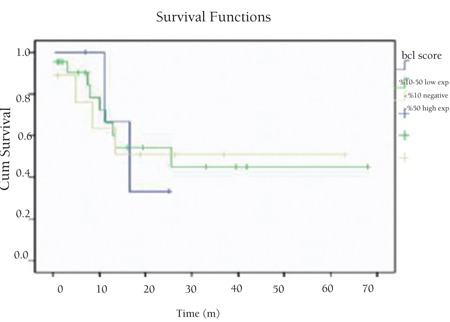
Overall survival related to Bcl-2 expression

There was no significant correlation between p53 and Bcl-2 expressions or Ki67 proliferation index and age, sex, stage, B symptoms, extranodal involvement, LDH level, and overall survival (p>0.05) (Table 2). We did not find significant relationships between p53, Bcl-2, and Ki67 expressions and prognosis (p>0.05) (Figures 5, 6, 7). There was no significant relationship between overall survival and age, sex, stage, B symptoms, extranodal involvement, or LDH level (p>0.05). 

Treatment records of 27 patients were attained. Twenty-three patients had been treated with 6-8 cycles of CHOP chemotherapy regimens including cyclophosphamide, doxorubicin, vincristine, and prednisolone. Only 4 patients had been treated with 6-8 cycles of a rituximab and CHOP chemotherapy regimen. Therefore, statistical analysis could not be done for the chemotherapy protocols. 

## DISCUSSION

DLBCL exhibits heterogeneous clinical features and varies markedly in response to treatment and prognosis [1,12]. Although survival can be estimated on the basis of clinical parameters, molecular abnormalities in a panel of suppressor proteins and oncogenic proteins have also been reported to be related to prognosis [5,6,7].

Several studies reported that age [7,12], serum LDH level [5,7,10,13], the involvement of extranodal sites [7,10], the stage of the disease [5,10,13,11,12,13,14,15], and B symptoms [10,15] were significant clinical predictors of survival of patients with DLBCL. In the present study, similar to the other studies, there was no statistically significant relationship between OS and age [5,11,13,14], sex [5,10,14,15], stage [16], B symptoms [13], extranodal involvement [11,13,15], or LDH level [11,15].

p53 can be considered as a tumor suppressor protein. It is involved in the regulation of cell survival by interaction with G-S phase transition within the cell cycle and by induction of apoptotic cell death [17,18]. The incidence of p53 expression in DLBCL varies between 5.0% and 71.0% [5,6,7,10,14,19,20,21,22]. In the present study, p53 expression was identified in 62.9% of all cases and high p53 expression was identified in 10 patients (28.6%). Although Ichikawa et al. reported that patients who had high p53 expression had increased LDH levels and advanced-stage disease [21], Sanchez et al. [5], Kramer et al. [6], Wilson et al. [19], and Rujirojindakul et al. [23] did not demonstrate statistical correlation between p53 expression and age, sex, LDH level, and B symptoms in patients with DLBCL. The present study failed to show a relationship between p53 protein expression and any of the clinical variables studied. Several studies reported a relationship between p53 expression and OS of patients with DLBCL [8,19,21]. Zhang et al. reported an inverse relationship between p53 expression and disease-free survival time [7]. Pervez et al. observed p53 nuclear expression in 52.13% of cases; it was concluded that p53 overexpression was associated with decreased OS (22). 

Piris et al. found a correlation between p53 expression and OS in high-grade lymphomas, and patients with combined expression of Bcl-2 and p53 in tumors had poorer prognosis than those with p53 expression only, which was particularly significant in lymph nodes in DLBCL cases [24]. Kramer et al. showed that p53 expression was only related to a high tumor burden and was not an independent risk factor for survival in patients with DLBCL [6]. Similarly, in the present study, no significant correlation was found between p53 expression and OS or disease-free survival [5,10,23]. It has been found that p53 expression had no significant effect on OS.

Bcl-2, an antiapoptotic protein, belongs to a large family of proteins involved in the regulation of programmed cell death [25]. The effects of Bcl-2 on clinical course have been widely studied with quite a few lymphoma types previously [5,6,7,10,13,19,20,26,27]. The incidence of Bcl-2 expression in DLBCL varies between 24.0% and 77.0% [5,6,7,10,13,20,22,24,26,27,28]. In the present study, 13 (37.1%) cases out of 35 showed Bcl-2 protein expression in tumor cells and 9 (25.7%) cases out of 35 exhibited high levels (>50.0% of tumor cells staining) of Bcl-2 protein expression. The relationship between Bcl-2 protein expression and clinical parameters has been evaluated previously. Bcl-2 protein expression has been found positive in patients with advanced disease stage [6,8,10,28], high LDH levels [27,29], and advanced age [8,20]. This study, similar to the other studies, failed to show a relationship between Bcl-2 protein expression and any of the clinical variables studied [6,10,19,26,28]. The prognostic significance of Bcl-2 protein expression is controversial [5,6,7,8,9,10,13,19,20,26,27]. Some studies [8,9,10,20,27,28,30] showed that Bcl-2 protein overexpression was associated with poor OS, but some other studies [5,6,7,13,19,26] showed no difference in OS. Pervez et al. reported positive Bcl-2 protein expression in 75 of 117 (64.1%) patients in their study on 117 patients with DLBCL. However, there was no significant difference in OS between patients with negative or weakly positive Bcl-2 and high Bcl-2 expressions [22]. Similarly, in the present study, we did not show any difference in the outcome of patients with Bcl-2-positive or Bcl-2-negative DLBCL. Iqbal et al. observed a relationship between Bcl-2 protein expression and survival in the ABC subgroup of DLBCL, which was not seen in the entire DLBCL group that was examined [29]. Different results were observed concerning the prognostic significance of Bcl-2 expression because of different cut-off values and methodological differences. 

Ki67 is a proliferation marker for several human neoplasms. Ki67 detects a nuclear antigen associated with cell proliferation [31]. The prognostic value of Ki67 has been the subject of different studies in the past. Although most of the studies showed a high proliferation index to be an adverse prognostic marker, there were inconsistent results, as well [32,33,34]. High Ki67 expression was observed in 23 patients (65.7%) in the present study. There was no correlation between Ki67 expression level and any studied clinicopathological factors. Miller et al. [32] reported that tumor proliferation of >80.0% was associated with poorer survival in previously untreated patients with aggressive NHL, whereas Hall et al. [35] found that patients who responded well to chemotherapy were less likely to relapse if they had >80.0% tumor proliferation. Jovanovic et al. reported that the patients with a high proliferative fraction (Ki67 > 60.0%) had a worse OS rate with log-rank test analysis [30]. Conversely, Hasselblom et al. found that patients with low Ki67 expression (<49.0%) had significantly worse progression-free survival and OS as independent risk factors [36]. In this study, similar to the other studies, there was no statistically significant relationship between Ki67 expression and prognosis [7,10,11,15]. 

The effects of p53 and Bcl-2 protein expressions and Ki67 proliferation index on prognosis of DLBCL have been investigated. The results from prior studies are variable and controversial. Methodological differences, different cut-off levels of immunohistochemical markers, and differences in the treatment regimens and follow-up time may account for the variation in results.

The present study revealed that Bcl-2 and p53 protein expressions and Ki67 proliferation index have no effect on the OS of patients with DLBCL. The prognostic importance of p53 and Bcl-2 protein expressions and Ki67 proliferation index in DLBCL, which has biological and clinical heterogeneity, can be understood in a large series of studies that have subclasses and immunohistochemical markers with optimal cut-off values.

## CONFLICT OF INTEREST STATEMENT

The authors of this paper have no conflicts of interest, including specific financial interests, relationships, and/ or affiliations relevant to the subject matter or materials included.

## Figures and Tables

**Table 1:**
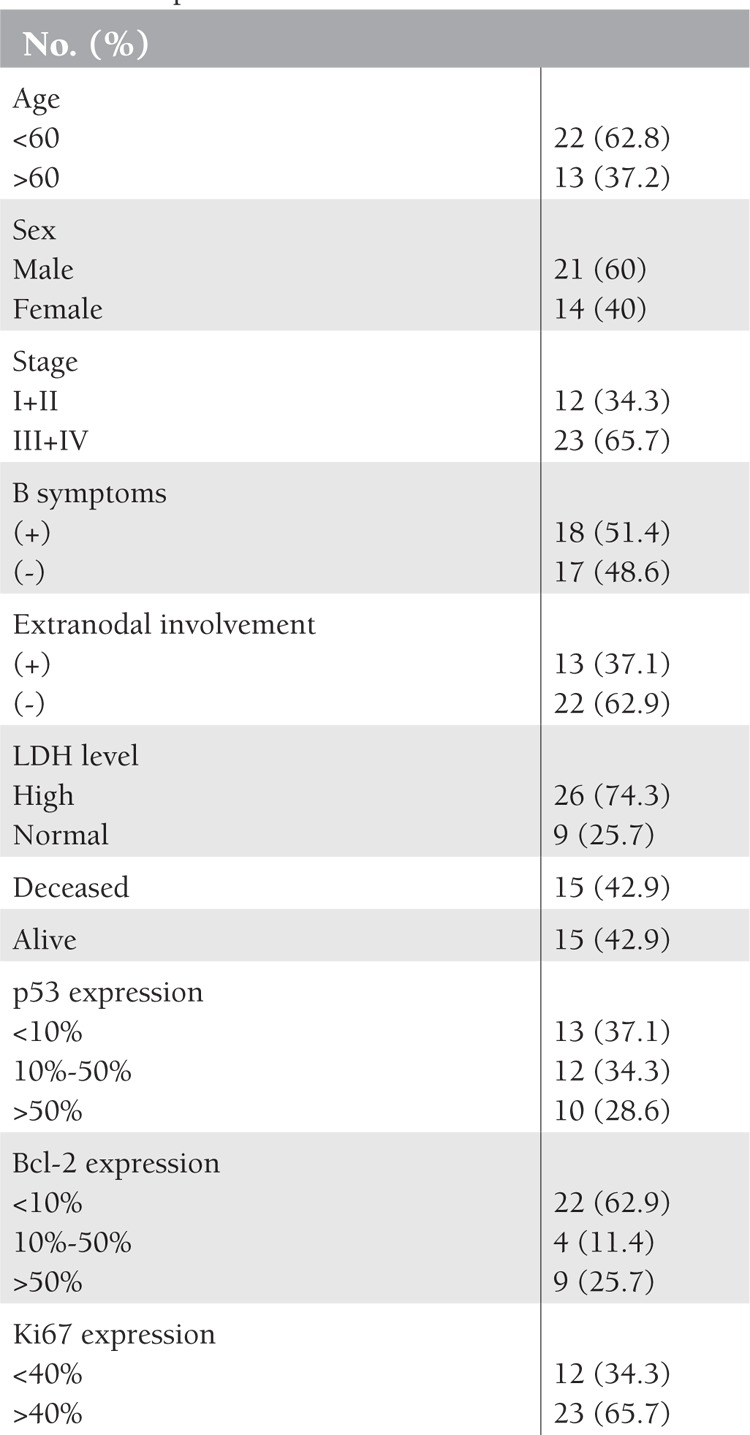
Characteristics of the patients and p53, Bcl-2,and Ki67 expressions

**Table 2:**
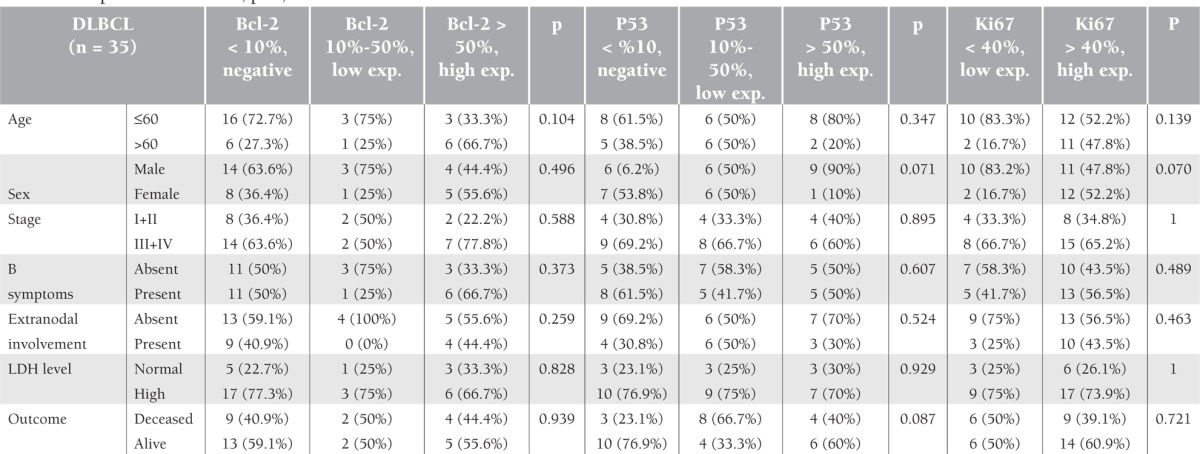
Expressions of Bcl-2, p53, and Ki67 in relation to clinical characteristics
